# Associação entre Tabagismo Passivo e Hipertensão: Um Estudo de Painel Com 621.506 Adultos do Brasil

**DOI:** 10.36660/abc.20250024

**Published:** 2025-05-19

**Authors:** Vicente Gabriel Winck Mattos, Gustavo Ianzer Moraes, Laís Werneck de Azevedo, Júlia Oleiro Mandeco, Elizabet Saes-Silva, Carine Nascimento da Silva, Samuel Carvalho Dumith

**Affiliations:** 1 Universidade Federal do Rio Grande Rio Grande RS Brasil Universidade Federal do Rio Grande, Rio Grande, RS – Brasil

**Keywords:** Hipertensão, Poluição por Fumaça de Tabaco, Brasil

## Abstract

**Fundamento:**

O tabagismo passivo (TP), que afeta um grande número de pessoas, pode criar uma predisposição para doenças cardiovasculares de forma semelhante ao tabagismo ativo. No entanto, essa relação é pouco explorada na literatura científica.

**Objetivos:**

Este estudo teve como objetivo investigar a associação entre TP e hipertensão em uma população adulta no Brasil.

**Métodos:**

Este estudo de painel utilizou dados da população brasileira coletados por meio de uma pesquisa VIGITEL realizada entre 2009 e 2021. Os dados foram analisados por meio de regressão de Poisson com intervalo de confiança de 95% (IC95%).

**Resultados:**

A prevalência de hipertensão em nossa população foi de 24,9% (IC95% 24,6-25,1), e o TP foi observado em 16,3% (IC95% 16,0-16,5). A análise ajustada revelou que o TP leva a um alto risco de hipertensão (RP=1,10; IC95% 1,07 a 1,14), que foi surpreendentemente próximo ao risco entre fumantes pesados (>1 maço ou 20 cigarros por dia) (RP 1,09; IC95% 1,06 a 1,13). Outro achado digno de nota foi a maior prevalência de hipertensão entre ex-fumantes, destacando associações que estão mal explicadas na literatura.

**Conclusão:**

Foi encontrada uma associação significativa entre TP e hipertensão, demonstrando que fumantes passivos são tão propensos a desenvolver hipertensão quanto fumantes pesados. Portanto, recomendamos uma meta-análise para consolidar as evidências sobre este assunto e fortalecer nossas descobertas.

## Graphical abstract

**Figura Central:**
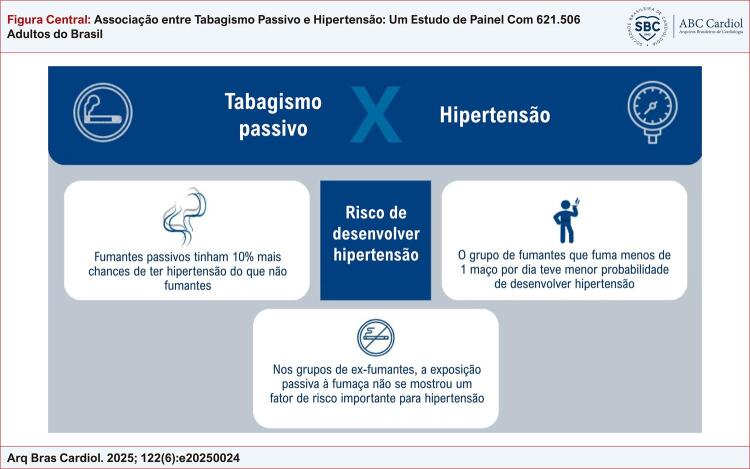
Associação entre Tabagismo Passivo e Hipertensão: Um Estudo de Painel Com 621.506 Adultos do Brasil

## Introdução

Tabagismo passivo (TP) se refere à inalação de fumaça de tabaco emitida por fumantes ativos por meio de dispositivos ou cigarros, seja em espaços fechados ou abertos. Desde 1990, a comunidade científica tem esclarecido o TP estudando suas possíveis implicações para a saúde e seu papel como fator de risco. Durante esse período, estudos examinaram a ligação entre o TP e condições como irritação nasofaríngea, câncer de pulmão e outras patologias.^1^ Embora a conexão entre o TP e doenças cardiovasculares (DCV) tenha sido amplamente investigada, um consenso científico ainda não foi estabelecido.^2,3^

No contexto da DCV, a pressão arterial alta (PAA) afeta aproximadamente 1,4 de bilhão de pessoas no mundo todo.^4^ Essa condição é definida pela pressão arterial sistólica (PAS) excedendo 140 mmHg ou pressão arterial diastólica (PAD) ultrapassando 90 mmHg, com medidas obtidas em pelo menos duas ocasiões distintas.^5^ Além de ser uma doença multifatorial, a progressão da PAA prejudica órgãos vitais. Ela eleva o risco de ataque cardíaco, derrame, doença renal crônica, cardiomiopatia, doenças coronárias, insuficiência cardíaca, retinopatia e outras DCV.^5^ Portanto, a hipertensão serve como um fator de risco para doenças fatais e é uma variável para prever morbidade e mortalidade.^4^ Além disso, estudos anteriores mostraram que a combinação de hipertensão e exposição ao tabaco - que é um fator de risco de DCV estabelecido por si só - pode aumentar os efeitos à saúde descritos acima.^6^

No contexto brasileiro, segundo o último relatório chamado Fatores de Risco e Proteção para Doenças Crônicas por Inquérito Telefônico (VIGITEL) (2021), a prevalência do diagnóstico de hipertensão na população é de 26,3%, e esse número vem crescendo anualmente. Por outro lado, a exposição ao tabaco entre adultos foi menor do que no ano anterior.^7^ As taxas de prevalência atuais são de 9,1% para tabagismo ativo, 6,9% para TP em casa e 5,4% para TP no ambiente de trabalho.^7^ Do ponto de vista financeiro, essa circunstância acarreta grandes gastos, pois, somente no Brasil, o ônus econômico direto do tabagismo ultrapassa R$ 50 bilhões (9,68 bilhões de dólares) a cada ano.^8^ Em comparação, as despesas com saúde relacionadas à hipertensão geralmente atingem uma média anual de R$ 8 bilhões (US$ 1,55 bilhão).^9^ No entanto, as perdas não são meramente econômicas: as mortes relacionadas a DCV correspondem a 50% e 25% do índice de mortalidade em países desenvolvidos e em desenvolvimento, respectivamente.^2^ Esses números ilustram o tremendo impacto do tabagismo e das DCV, que levam a perdas financeiras, sociais e humanas.

Embora poucos estudos tenham associado TP e DCV,^2^ há uma lacuna ainda maior no estabelecimento de uma relação causal entre TP e DCV. Portanto, se essa associação for estatisticamente significativa, a combinação deste estudo com outras pesquisas estatisticamente potentes pode ser útil para o planejamento administrativo dos sistemas de saúde, uma vez que a definição de um novo fator de risco modificável possibilita a implementação de políticas de saúde direcionadas. Assim, por meio da análise de dados de 2009 a 2021 extraídos do inquérito VIGITEL, este estudo teve como objetivo investigar a relação entre hipertensão e TP em populações adultas e idosas brasileiras.

## Métodos

Este estudo de painel utilizou dados da pesquisa VIGITEL^7^ realizada entre 2009 e 2021. Resumidamente, o VIGITEL é uma pesquisa de saúde complexa que visa monitorar a frequência e a distribuição de doenças crônicas não transmissíveis (DCNT) nas capitais de todos os 26 estados brasileiros e no Distrito Federal. Desde seu início em 2006 até 2021, um total de 784.479 brasileiros foram entrevistados. Os dados foram coletados por meio de questionários administrados por telefone. Mais detalhes sobre o processo metodológico e a coleta de dados são fornecidos no relatório da pesquisa.^7^

O consentimento livre e esclarecido foi obtido oralmente durante ligações telefônicas. O Comitê Nacional de Ética em Pesquisa em Seres Humanos do Ministério da Saúde aprovou a pesquisa VIGITEL (CAAE;65610017.1.0000.0008).^7^

De 2006 a 2019, a pesquisa teve um tamanho amostral mínimo entre 1500 e 2000 indivíduos em cada cidade.^7^ No entanto, devido à pandemia de COVID-19 em 2020 e 2021, o período de coleta de dados foi limitado ao primeiro trimestre de cada ano (janeiro a abril), reduzindo a amostra mínima para 1000 indivíduos por cidade. Em ambas as configurações, o tamanho amostral permitiu uma estimativa com intervalo de confiança de 95% (IC95%) e erro máximo de quatro pontos percentuais da frequência dos fatores de risco e proteção analisados na população adulta.

Os dados relacionados à hipertensão foram obtidos com base em um diagnóstico médico prévio desta doença, conforme indicado pela resposta à pergunta: “Algum médico já lhe disse que você tem pressão alta?”. O desfecho foi estabelecido de forma dicotômica (sim ou não).

Além disso, dados sobre tabagismo e TP (variáveis incluídas em 2009) relacionados a este estudo foram obtidos usando as perguntas descritas abaixo. A porcentagem de fumantes passivos em casa e no trabalho foi obtida por ex-fumantes ou nunca fumantes que responderam “Sim” a uma destas perguntas: “Alguma das pessoas que moram com você costuma fumar dentro de casa?” ou “Algum dos seus colegas de trabalho costuma fumar no mesmo local onde você trabalha?”. Enquanto isso, fumantes ativos foram definidos como indivíduos que responderam “Sim, diariamente” à pergunta “Você fuma atualmente?”, independentemente do número de cigarros fumados ou da duração do hábito de fumar. Se a resposta fosse afirmativa, o número de cigarros fumados por dia era registrado. O número de ex-fumantes foi definido pela resposta “Sim, diariamente” à pergunta “Você já fumou no passado?”. Indivíduos que nunca fumaram ou que eram fumantes ocasionais foram categorizados com base em suas respostas negativas às perguntas anteriores. Para este estudo, todas as respostas foram registradas como dicotômicas (sim ou não), e a carga tabágica foi agrupada de acordo com o consumo de mais ou menos 20 cigarros (um maço) por dia.

Para fins de análise estatística e discussão, os não fumantes foram estabelecidos como grupo controle, e os participantes foram divididos em seis estratos: nunca fumante e fumante não passivo, nunca fumante e fumante passivo, ex-fumante e fumante não passivo, ex-fumante e fumante passivo, fumante leve atual (< 1 maço/dia) e fumante pesado atual (≥1 maço/dia).

As seguintes variáveis foram incluídas na descrição da amostra para diminuir fatores de confusão: sexo (masculino ou feminino), idade (18-39; 40-59 e 60 ou mais), cor (branca, preta, parda, asiática, indígena), escolaridade (0-8; 9-11; 12 ou mais anos de estudo), estado civil (solteiro, casado, em união estável, viúvo ou divorciado), morar sozinho (sim ou não) e região de residência (Norte, Nordeste, Centro-Oeste, Sudeste ou Sul).

A análise dos dados foi realizada com a versão 15.1 do software estatístico Stata^®^, utilizando a ferramenta “*persorake*” para contabilizar o peso da pesquisa do VIGITEL.^7^ Inicialmente, realizamos uma análise univariada para descrever as frequências absolutas e relativas da amostra. Posteriormente, a prevalência da análise do resultado foi calculada usando um teste qui-quadrado. As análises brutas e ajustadas foram realizadas usando regressão de Poisson com ajuste robusto de variância, intervalos de confiança de 95% e valor de p. Um nível de significância de 0,05 foi usado para todas as análises.

## Resultados

No geral, analisamos 621.506 adultos (com 18 anos ou mais) que foram entrevistados entre 2009 e 2021. A idade média foi de 49,3 anos (DP=18,1), e houve maior frequência de mulheres (54%). Além disso, indivíduos de etnia negra representaram 45,7% da população do estudo, indivíduos com 9 a 11 anos de escolaridade 37,7%, solteiros 40,6%, vivendo com uma ou mais pessoas 96,8%, moradores da região sudeste 45,1%. Além disso, 56,1% dos indivíduos nunca fumaram. Considerando toda a amostra, 24,85% (IC95% 24,6-25,1) dos indivíduos foram identificados como hipertensos. Essas informações estão resumidas na Tabela 1.

**Tabela 1:** Características da população no período de 2009 a 2021 (n= 621.506) segundo a pesquisa VIGITEL, Brasil

Variável	Não	%
**Sexo**		
Masc.	286 139	46,0
Fem.	335 367	54,0
**Faixa etária**		
18-39	306 731	49,4
40-59	209 757	33,7
60 ou mais	105 018	16,9
**Cor da pele**		
Branco	253 197	43,3
Preto	266 916	45,7
Amarelo	56 638	9,7
Indígena	7 598	1,3
**Escolaridade (em anos)**		
0 a 8 anos	210 217	33,8
9 a 11 anos	234 021	37,7
12 anos ou mais	177 268	28,5
**Estado civil**		
Solteiro	250 026	40,6
Casado	232 337	37,7
União estável	66 291	10,8
Viúvo	31 440	5,1
Separado ou divorciado	35 729	5,8
**Mora sozinho**		
Não	601 758	96,8
Sim	19 748	3,2
**Região**		
Norte	62 691	10,1
Nordeste	156 513	25,2
Centro-Oeste	71 672	11,5
Sudeste	280 208	45,1
Sul	50 422	8,1
**Tabagismo**		
Nunca fumante e fumante não passivo	348 429	56,1
Nunca fumante e fumante passivo	103 411	16,6
Ex-fumante e fumante não passivo	74 801	12,0
Ex-fumante e fumante passivo	26 267	4,2
Fumante leve atual (<1 maço/dia)	55 263	8,9
Fumante inveterado atual (≥1 maço/dia)	13 335	2,2
% Prevalência		

**Figura 1:**
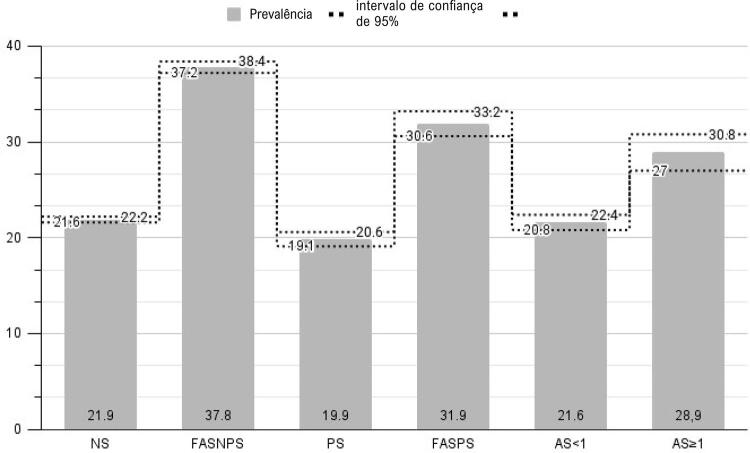
Prevalência e intervalo de confiança de 95%. Prevalência de hipertensão por cada estrato de tabagismo entre 2009 e 2021 (n= 621.506) segundo dados do VIGITEL, com intervalo de confiança de 95%. NS: nunca fumante e fumante não passivo; FASNPS: ex-fumante e fumante não passivo; PS: nunca fumante e fumante passivo; FASPS: ex-fumante e fumante passivo; AS<1: Fumante leve atual (< 1 maço/dia); AS≥ 1: Fumante pesado atual (≥ 1 maço/dia).

A análise da prevalência de PAA de acordo com o estrato de tabagismo mostrou que a maior prevalência foi de 37,8%, encontrada entre ex-fumantes que não eram fumantes passivos. Além disso, 19,9% dos fumantes passivos tinham hipertensão, o que foi semelhante à prevalência de 21,6% encontrada entre fumantes leves atuais. Ex-fumantes ativos tiveram uma prevalência de PAA de 31,9%, o que foi muito semelhante à encontrada entre fumantes pesados atuais (28,9%). Enquanto isso, a prevalência de hipertensão entre nunca fumantes que eram fumantes não passivos foi de 21,9%. Os dados são mostrados na Figura 1 e na Ilustração Central.

Da análise bruta dos seis estratos descritos anteriormente, ser ex-fumante ativo que não é fumante passivo (RP 1,72; IC95% 1,64;1,81; p<0,001), ser ex-fumante ativo que é fumante passivo (RP 1,45; IC95% 1,40;1,51; p<0,001) e ser fumante ativo que fuma mais de um maço por dia (RP 1,32; IC95% 1,24;1,40; p<0,001) são fatores de risco para hipertensão. Enquanto isso, o grupo que fumava menos de 1 maço por dia (RP 0,98; IC95% 0,95;1,02) não foi associado à hipertensão, e os grupos de nunca fumantes e fumantes passivos foram menos propensos a ter hipertensão (RP 0,91; IC95% 0,89;0,92; p<0,001).

De acordo com as análises bruta e ajustada (Tabela 2), os nunca fumantes que eram fumantes passivos atuais apresentaram um efeito de confusão paradoxal, sendo um fator de proteção na análise bruta, mas um fator de risco para hipertensão na análise ajustada. A magnitude desse efeito foi semelhante à dos fumantes pesados e maior do que a do grupo de fumantes leves, mesmo após o ajuste. Para ex-fumantes, o TP não alterou os efeitos da hipertensão.

**Tabela 2:** – Análise do modelo de regressão de Poisson para hipertensão entre fumantes brasileiros de 2009 a 2021 (n= 621.506), inquérito VIGITEL, Brasil

Variáveis independentes	Análise Bruta			Análise Ajustada*		
	Medida de efeito RP	IC95%	Valor p	Medida de efeito RP	IC95%	Valor p
Fumar			<0,001			<0,001
Nunca fumante e fumante não passivo	1,00	-		1,00	-	
Ex-fumante e fumante não passivo	1,72	1,64;1,81		1,16	1,14;1,19	
Nunca fumante e fumante passivo	0,91	0,89;0,92		1,10	1,07;1,14	
Ex-fumante e fumante passivo	1,45	1,40;1,51		1,17	1,14;1,20	
Fumante leve atual (< 1 maço/dia)	0,98	0,95;1,02		0,93	0,90;0,95	
Fumante inveterado atual (≥1 maço/dia)	1,32	1,24;1,40		1,09	1,06;1,13	

*Ajustado para cidades do cluster 27. Ajuste para fatores de confusão: sexo, idade, cor da pele, escolaridade, estado civil, mora sozinho e região. RP: razão de prevalência; IC95%: intervalo de confiança de 95%.

## Discussão

Este estudo teve como objetivo determinar a associação entre TP e hipertensão. Analisamos dados de fumantes brasileiros, ativos e passivos, de 2009 a 2021, usando nunca fumantes e fumantes não passivos como grupo controle. Na análise ajustada, o grupo nunca fumante e fumante passivo teve uma probabilidade 10% (IC95% 1,07 a 1,14) maior de desenvolver hipertensão, que foi semelhante aos 9% (IC95% 1,06 a 1,13) do grupo de fumantes pesados atuais (≥ 1 maço/dia). Esses resultados estão alinhados com os de pesquisas anteriores,^10-13^ pois mostram uma associação significativa entre hipertensão e o grupo de nunca fumantes e fumantes passivos. Além disso, encontramos probabilidades semelhantes de hipertensão para os grupos de ex-fumantes e fumantes não passivos e ex-fumantes e fumantes passivos (16% e 17% maiores, respectivamente), o que indica que a exposição passiva ao tabaco tem uma influência limitada no risco de hipertensão para ex-fumantes.

Com base na análise ajustada da associação entre hipertensão e os vários estratos de exposição ao tabaco, observamos riscos muito semelhantes para o grupo de nunca fumantes e fumantes passivos e o fumante pesado atual (≥1 maço/dia). Este achado demonstra os efeitos nocivos do TP entre os nunca fumantes, que, neste estudo, mostraram ser equivalentes aos dos fumantes pesados atuais (≥1 maço/dia). Uma hipótese para esse achado controverso está nas diferentes composições químicas da fumaça inalada por fumantes passivos,^3^ chamada de fumaça lateral. Sua toxicidade, menor do que a encontrada em situações cotidianas quando medida em concentrações atmosféricas, costuma ser quatro vezes maior do que a da fumaça convencional,^14^ ou seja, a fumaça inalada por fumantes ativos. Vale ressaltar que fumantes ativos também são expostos à fumaça lateral, o que poderia inicialmente sustentar a hipótese de maior potencial de dano em comparação ao TP. No entanto, se deve ressaltar que, a longo prazo, esse grupo tem maior exposição aos efeitos hipotensores da nicotina do que fumantes passivos, que inalam menos nicotina. Essa diferença fornece uma explicação plausível para as medidas de efeito semelhantes.^15,16^ Portanto, concluímos que fundamentos fisiopatológicos justificam a semelhança encontrada entre esses dois grupos.

Em relação à análise ajustada, o consumo de menos de 20 cigarros por dia, que foi classificado como o grupo de fumantes leves atuais (< 1 maço/dia), foi considerado um fator de proteção contra hipertensão em comparação ao grupo de nunca fumantes e fumantes não passivos. Este resultado se soma a uma série de achados controversos sobre a relação entre exposição ao tabaco e hipertensão, com vários estudos demonstrando níveis mais baixos de pressão arterial (PA) ou uma maior prevalência de hipertensão mascarada entre fumantes (16% e 17%, respectivamente). A hipótese fisiopatológica para esse fenômeno, como a adaptação do corpo à nicotina e seus metabólitos, que levam a uma redução inicial da PA, já foi estudada por alguns autores; no entanto, o mecanismo permanece obscuro.^10-15^ Portanto, levantamos a hipótese de que o efeito protetor do tabagismo leve é uma consequência da exposição à nicotina. Como mencionado anteriormente, e em contraste com os efeitos pró-hipertensivos do monóxido de carbono, a nicotina promove hipotensão, um efeito que só é detectável após anos de exposição intensa.^15^

Vale ressaltar que estudos anteriores demonstraram um aumento proporcional no risco de hipertensão de acordo com a magnitude da exposição ao tabaco, seja medida em tempo ou quantidade de cigarros.^17,18^Esses dados contrastam a influência não linear do número de cigarros na PA de fumantes ativos, conforme descrito neste artigo. Portanto, enfatizamos o impacto variável da exposição ao tabaco na PA e sua dependência da forma de exposição (ativa ou passiva).

Outro resultado interessante foi a medida do efeito da hipertensão em grupos de ex-fumantes em comparação com os fumantes pesados atuais (≥1 maço/dia). Esses dados corroboram os achados de pesquisas anteriores, que, além de mostrar uma ligação entre maiores cargas de tabaco e menores níveis de PA, demonstraram que o tabagismo regular estava significativamente associado a menores PAS e PAD. Em contraste, a cessação do tabagismo estava associada a maiores níveis de PAD em ambos os grupos em comparação aos nunca fumantes. Entre as possíveis explicações para o maior risco de hipertensão em ex-fumantes, uma das hipóteses atribui o aumento da PA ao ganho de peso que geralmente ocorre após a cessação do tabagismo.^19-21^ Essa teoria é provavelmente a mais amplamente aceita, especialmente porque o ganho de peso em si está entre os principais fatores de risco para hipertensão.^22^ Além disso, há duas outras possíveis razões para esse fenômeno, embora não tão fortemente apoiadas pela literatura: a hipótese de hipertensão mascarada em fumantes ativos e causalidade reversa.^16,23^

Por fim, vale ressaltar que os mecanismos fisiopatológicos da nicotina e seus metabólitos, bem como a influência do monóxido de carbono no ciclo do óxido nítrico e os danos cardiovasculares globais causados pela fumaça do tabaco, que são cruciais para sustentar nossos achados, já foram amplamente explicados na literatura científica.^3,6^

A principal limitação do nosso estudo é o seu delineamento transversal, o que impediu o estabelecimento de uma relação causal entre o grupo de nunca fumantes e fumantes passivos e a hipertensão. Assim, apesar de encontrar associações significativas, não conseguimos confirmar uma relação causal entre essas variáveis, sendo, portanto, essencial a realização de novos estudos longitudinais para explorar essa associação na população brasileira.

Além disso, a dependência deste estudo na pesquisa VIGITEL também é uma limitação devido à natureza autorrelatada das informações, o que pode levar a subestimações em comparação com métodos como análise de cotinina sérica e medições de pressão arterial. Além disso, existem outras limitações para este estudo, como não questionar a duração da exposição dos participantes ao tabaco, anos desde a cessação do tabagismo, gravidade da hipertensão, anos desde o diagnóstico de hipertensão e prescrição de anti-hipertensivos. Esses dados podem aumentar nossa compreensão de como o TP influencia o desenvolvimento e a progressão da hipertensão.^24^ Além disso, como já mencionado na seção de metodologia, a pandemia de COVID-19 durante 2020 e 2021 restringiu o prazo da pesquisa, reduzindo assim o tamanho da amostra.

Entre os pontos fortes do estudo está o fato de que este estudo utilizou uma sólida base de dados nacional focada na investigação de DCNT, incluindo dados de todas as capitais dos estados e do Distrito Federal, com uma amostra de mais de 600 mil pessoas. Até onde sabemos, este é o maior estudo no Brasil sobre este assunto e o primeiro a utilizar uma amostra nacional de base populacional. Acreditamos que nossos achados podem ser extrapolados para outros países devido à plausibilidade biológica das associações analisadas. Além disso, nossos dados podem ser usados para implementar políticas sobre comportamento de fumar em ambientes fechados.

## Conclusão

No geral, este estudo encontrou uma associação estatisticamente significativa entre TP e hipertensão. Além disso, foi feita uma comparação entre a prevalência de hipertensão em fumantes passivos e ativos. Por fim, destacamos a importância do desenvolvimento de mais estudos longitudinais sobre este tópico, visando acompanhamentos prolongados e direcionados para compreender melhor as alterações da PA causadas pelo TP. Este conhecimento é necessário para promover a prevenção eficaz e a redução de danos no contexto do tabagismo e do TP.
