# Supplementary material for: Associação entre Tabagismo Passivo e Hipertensão: Um Estudo de Painel Com 621.506 Adultos do Brasil
Source: Arq Bras Cardiol. 2025 May 19;122(6):e20250024. [Article in Portuguese] doi: 10.36660/abc.20250024 (PMC12133067; doi:10.36660/abc.20250024)
Supplement: Translation

# Translation: Association between Passive Smoking and Hypertension: A Panel Study with 621.506 Adults from Brazil

**DOI:** 10.36660/abc.20250024i

**Authors:** Vicente Gabriel Winck Mattos, Gustavo Ianzer Moraes, Laís Werneck de Azevedo, Júlia Oleiro Mandeco, Elizabet Saes-Silva, Carine Nascimento da Silva, Samuel Carvalho Dumith

**Affiliations:** 1 Brazil Universidade Federal do Rio Grande, Rio Grande, RS – Brazil

**Keywords:** Hypertension, Tobacco Smoke Pollution, Brazil

## Abstract

**Background:**

Passive smoking, which affects a large number of people, may create a predisposition to cardiovascular disease in a manner similar to active smoking. However, this relationship is poorly explored in the scientific literature.

**Objectives:**

This study aimed to investigate the association between passive smoking and hypertension in an adult population in Brazil.

**Methods:**

This panel study utilized data on the Brazilian population collected through a VIGITEL survey conducted between 2009 and 2021. The data were analyzed using Poisson regression with a 95% confidence interval (95%CI).

**Results:**

The prevalence of hypertension in our population was 24.9% (95%CI 24.6-25.1), and passive smoking was observed in 16.3% (95%CI 16.0-16.5). The adjusted analysis revealed that passive smoking leads to a high risk of hypertension (PR=1.10; 95%CI 1.07 to 1.14), which was surprisingly close to the risk among heavy smokers (>1 pack or 20 cigarettes a day) (PR 1.09; 95%CI 1.06 to 1.13). Another noteworthy finding was the higher prevalence of hypertension among former smokers, highlighting associations that are poorly explained in the literature.

**Conclusion:**

A significant association was found between passive smoking and hypertension, demonstrating that passive smokers are as prone to developing hypertension as heavy smokers. Therefore, we recommend a meta-analysis to consolidate the evidence on this subject and strengthen our findings.

## Introduction

Second-hand smoke (SHS) and passive smoke (PS) refer to the inhalation of tobacco smoke emitted by active smokers through devices or cigarettes, whether in closed or open spaces. Since 1990, the scientific community has shed light on PS by studying its possible health implications and its role as a risk factor. During this period, studies have examined the link between PS and conditions such as nasopharyngeal irritation, lung cancer, and other pathologies.^1^ Although the connection between PS and cardiovascular diseases (CVD) has been extensively investigated, a scientific consensus has not yet been established.^2,3^

In the context of CVD, high blood pressure (HBP) affects approximately 1.4 billion people worldwide.^4^ This condition is defined by systolic blood pressure (SBP) exceeding 140 mmHg or diastolic blood pressure (DBP) surpassing 90 mmHg, with measures obtained on at least two separate occasions.^5^ In addition to being a multifactorial disease, HBP progression harms vital organs. It elevates the risk for heart attack, stroke, chronic renal disease, cardiomyopathy, coronary diseases, heart failure, retinopathy, and other CVD.^5^ Therefore, hypertension serves as both a risk factor for life-threatening illnesses and a variable for predicting morbidity and mortality.^4^ Furthermore, previous studies have shown that the combination of hypertension and tobacco exposure - which is an established CVD risk factor on its own - can escalate the health effects described above.^6^

In the Brazilian context, according to the latest Brazilian Protective and Risk Factors for Chronic Diseases by Telephone Survey (VIGITEL) report (2021), the prevalence of hypertension diagnosis in the population stands at 26.3%, and this figure has been growing annually. Conversely, tobacco exposure among adults was lower than in the previous year.^7^ The current prevalence rates are 9.1% for active smoking, 6.9% for passive smoking at home, and 5.4% for passive smoking in the workplace.^7^ From a financial perspective, this circumstance leads to major expenses since, only in Brazil, the direct economic burden of smoking exceeds 50 billion BRL (9.68 billion dollars) each year.^8^ In comparison, the healthcare expenses related to hypertension usually average a yearly 8 billion BRL (1.55 billion dollars).^9^ However, the losses are not merely economic: CVD-related deaths correspond to 50% and 25% of the mortality index in developed and developing countries, respectively.^2^ These numbers illustrate the tremendous impact of smoking and CVD, which lead to financial, social, and human losses.

Although few studies have associated PS and CVD,^2^ there is an even larger gap in the establishment of a causal relationship between PS and CVD. Therefore, if this association is statistically significant, the combination of this study with other statistically powerful research may be useful for the administrative planning of health systems, given that defining a new modifiable risk factor makes it possible to implement targeted health policies. Thus, by analyzing data from 2009 to 2021 extracted from the VIGITEL survey, this study aimed to investigate the relationship between hypertension and passive smoking in Brazilian adult and elderly populations.

## Methods

This panel study used data from the VIGITEL^7^ survey conducted between 2009 and 2021. Briefly, VIGITEL is a complex health survey aimed at monitoring the frequency and distribution of non-communicable chronic diseases (NCD) in the capitals of all 26 Brazilian states and the Federal District. Since its inception in 2006 up until 2021, a total of 784.479 Brazilians have been interviewed. Data were collected through phone-administered questionnaires. Further details on the methodological process and data collection are provided in the survey report.^7^

Free and informed consent was obtained orally during phone calls. The National Committee of Ethics in Research for Human Beings of the Ministry of Health approved the VIGITEL survey(CAAE;65610017.1.0000.0008).^7^

From 2006 to 2019, the survey had a minimum sample size of between 1500 and 2000 individuals in each city.^7^ However, owing to the COVID-19 pandemic in 2020 and 2021, the data collection period was limited to the first quarter of each year (January to April), reducing the minimum sample to 1000 individuals per city. In both configurations, the sample size allowed an estimation with a 95% confidence interval (95%CI) and a maximum error of four percentage points of the frequency of the risk and protective factors analyzed in the adult population.

Data related to hypertension were obtained based on a previous medical diagnosis of this disease, as indicated by the response to the question, “Has any doctor ever told you that you have high blood pressure?”. The outcome was established dichotomously (yes or no).

In addition, smoking and passive smoking (variables included in 2009) data related to this study were obtained using the questions described below. The percentage of passive smokers at home and work was obtained by former or never smokers who answered “Yes” to one of these questions: “Do any of the people who live with you usually smoke inside the house?” or “Do any of your coworkers usually smoke in the same place where you work?”. Meanwhile, active smokers were defined as individuals who answered “Yes, daily” to the question “Do you currently smoke?” regardless of the number of cigarettes smoked or the duration of the smoking habit. If the answer was affirmative, the number of cigarettes smoked per day was recorded. The number of former smokers was defined by the answer “Yes, daily” to the question “Have you ever smoked in the past?”. Individuals who had never smoked or who were occasional smokers were categorized based on their negative answers to the previous questions. For this study, all answers were registered as dichotomous (yes or no), and the smoking load was grouped according to the consumption of more or less than 20 cigarettes (one pack) per day.

For statistical analysis and discussion purposes, nonsmokers were established as the control group, and participants were divided into six strata: never-smoker and nonpassive smoker, never-smoker and passive smoker, former smoker and nonpassive smoker, former smoker and passive smoker, current light smoker (< 1 pack/day) and current heavy smoker (≥ 1 pack/day).

The following variables were included in the sample description to diminish confounding factors: sex (male or female), age (18-39; 40-59 and 60 or more), color (white, black, brown, Asian, indigenous), schooling (0-8; 9-11; 12 or more years of study), marital status (single, married, in common law marriage, widowed, or divorced), living alone (yes or no), and region of residence (North, Northeast, Midwest, Southeast, or South).

The data analysis was performed with the 15.1 version of Stata^®^ statistical software, using the tool “persorake” to account for VIGITEL’s survey weight.^7^ Initially, we performed a univariate analysis to describe the sample’s absolute and relative frequencies. Subsequently, the prevalence of the endpoint analysis was calculated using a chi-square test. Crude and adjusted analyses were performed using Poisson regression with robust adjustment of variance, 95% confidence intervals, and *p*-value. A significance level of 0.05 was used for all analyses.

## Results

Overall, we analyzed 621,506 adults (aged 18 years or older) who were surveyed between 2009 and 2021. The average age was 49.3 years old (DP=18.1), and there was a higher frequency of females (54%). Further, individuals of black ethnicity accounted for 45.7% of the study population, individuals with 9–11 years of schooling 37.7%, singles for 40.6%, living with one or more people for 96.8%, southeast region residents for 45.1%. Additionally, 56.1% of individuals had never smoked. Considering the entire sample, 24.85% (95%CI 24.6-25.1) of individuals were identified as being hypertensive. This information is summarized in Table 1.

**Table 1:** – Characteristics of the population in the period between 2009 and 2021 (n= 621,506) according to the VIGITEL survey, Brazil

Variable	N	%
**Sex**		
Male	286 139	46.0
Female	335 367	54.0
**Age group**		
18-39	306 731	49.4
40-59	209 757	33.7
60 or more	105 018	16.9
**Skin color**		
White	253 197	43.3
Black	266 916	45.7
Yellow	56 638	9.7
Indigenous	7 598	1.3
**Schooling (in years)**		
0 to 8 years	210 217	33.8
9 to 11 years	234 021	37.7
12 years or more	177 268	28.5
**Marital status**		
Single	250 026	40.6
Married	232 337	37.7
Common law marriage	66 291	10.8
Widower	31 440	5.1
Separated or divorced	35 729	5.8
**Lives alone**		
No	601 758	96.8
Yes	19 748	3.2
**Region**		
North	62 691	10.1
Northeast	156 513	25.2
Midwest	71 672	11.5
Southeast	280 208	45.1
South	50 422	8.1
**Smoking**		
Never-smoker and nonpassive smoker	348 429	56.1
Never-smoker and passive smoker	103 411	16.6
Former smoker and nonpassive smoker	74 801	12.0
Former smoker and passive smoker	26 267	4.2
Current light smoker (< 1 pack/day)	55 263	8.9
Current heavy smoker (≥ 1 pack/day)	13 335	2.2
% Prevalence		

Analysis of the prevalence of HBP according to the smoking stratum showed that the highest prevalence was 37.8%, found among former smokers who were not passive smokers. Furthermore, 19.9% of passive smokers had hypertension, which was similar to the 21.6% prevalence found among current light smokers. Former active smokers had an HBP prevalence of 31.9%, which was very similar to that found among current heavy smokers (28.9 %). Meanwhile, the prevalence of hypertension among never-smokers who were nonpassive smokers was 21.9%. The data are shown in the Figure 1 and the Central Illustration*.*

**Figure 1:** – Prevalence and 95% Confidence interval. Prevalence of hypertension per each smoking strata between 2009 and 2021 (n= 621,506) according to VIGITEL’s data, with a 95% confidence interval. NS: never-smoker and nonpassive smoker; FASNPS: former smoker and nonpassive smoker; PS: never-smoker and passive smoker; FASPS: Former smoker and passive smoker; AS<1: Current light smoker (< 1 pack/day); AS≥ 1: Current heavy smoker (≥ 1 pack/day).

From the crude analysis of the six previously described strata, being a former active smoker who is not a passive smoker (PR 1.72; 95%CI 1.64;1.81, p<0.001), being a former active smoker who is a passive smoker (PR 1.45; 95%CI 1.40;1.51; p<0.001) and being an active smoker who smokes more than one pack a day (PR 1.32; 95%CI 1.24;1.40; p<0.001) are risk factors for hypertension. Meanwhile, the group smoking less than 1 pack a day (PR 0.98; 95%CI 0.95;1.02) was not associated with hypertension, and the never-smoker and passive-smoker groups were less likely to have hypertension (PR 0,91; 95%CI 0,89;0,92; p<0,001).

According to the crude and adjusted analyses (Table 2), never-smokers who were current passive smokers presented a paradoxical confounding effect, being a protective factor in the crude analysis but a risk factor for hypertension in the adjusted analysis. The magnitude of this effect was similar to that of heavy smokers and higher than that of the light smoker group, even after adjustment. For former smokers, passive smoking did not alter the effects of hypertension.

**Table 2:** – Analysis of the Poisson Regression model for hypertension among Brazilian smokers from 2009 to 2021 (n= 621,506), VIGITEL survey, Brazil

Independent variables	Crude Analysis			Adjusted Analysis*		
	Effect measure PR	95%CI	p value	Effect measure PR	95%CI	p value
**Smoking**			<0.001			<0.001
Never-smoker and nonpassive smoker	1.00	-		1.00	-	
Former smoker and nonpassive smoker	1.72	1.64;1.81		1.16	1.14;1.19	
Never-smoker and passive smoker	0.91	0.89;0.92		1.10	1.07;1.14	
Former smoker and passive smoker	1.45	1.40;1.51		1.17	1.14;1.20	
Current light smoker (< 1 pack/day)	0.98	0.95;1.02		0.93	0.90;0.95	
Current heavy smoker (≥ 1 pack/day)	1.32	1.24;1.40		1.09	1.06;1.13	

*Adjusted for cluster 27 cities. Adjustment for confounding factors: sex, age, skin color, schooling, marital status, lives alone, and region. PR: prevalence ratio; 95% CI: 95% confidence interval.

## Discussion

This study aimed to determine the association between passive smoking and hypertension. We analyzed data on Brazilian smokers, both active and passive, from 2009 to 2021, using never-smokers and nonpassive smokers as the control group. On the adjusted analysis, the never-smoker and passive smoker group had a 10% (95%CI 1.07 to 1.14) higher probability of developing hypertension, which was similar to the current heavy smoker (≥ 1 pack/day) group’s 9% (95%CI 1.06 to 1.13). These results are aligned with those of previous research,^10-13^ as they show a significant association between hypertension and the never-smoker and passive-smoker group. Furthermore, we found similar hypertension probabilities for the former smoker and nonpassive smoker and former smoker and passive smoker groups (16% and 17% higher, respectively), which indicates that passive tobacco exposure has a limited influence on hypertension risk for former smokers.

Based on the adjusted analysis of the association between hypertension and the various tobacco exposure strata, we observed very similar risks for the never-smoker and passive smoker group and the current heavy smoker (≥ 1 pack/day) group. This finding demonstrates the harmful effects of passive smoke among never-smokers, who, in this study, were shown to be equivalent to those of current heavy smokers (≥ 1 pack/day). A hypothesis for this controversial finding lies in the different chemical compositions of smoke inhaled by passive smokers,^3^ called sidestream smoke. Its toxicity, which is lower than that found in day-to-day situations when measured at atmospheric concentrations, is usually four times greater than that of mainstream smoke,^14^ that is, the smoke inhaled by active smokers. It is worth noting that active smokers are also exposed to sidestream smoke, which could initially support the hypothesis of greater harm potential compared to that of passive smoking. However, it must be noted that, in the long term, this group has greater exposure to the hypotensive effects of nicotine than passive smokers, who inhale less nicotine. This difference provides a plausible explanation for the similar effect measures.^15,16^ Therefore, we conclude that physiopathological foundations justify the similarity found between these two groups.

Regarding the adjusted analysis, consumption of less than 20 cigarettes a day, which was classified as the current light smoker (< 1 pack/day) group, was found to be a protective factor against hypertension in comparison to the never-smoker and nonpassive smoker group. This result adds to a series of controversial findings regarding the relationship between tobacco exposure and hypertension, with various studies demonstrating either lower blood pressure (BP) levels or a higher prevalence of masked hypertension among smokers (16% and 17%, respectively). The physiopathological hypothesis for this phenomenon, such as the body’s adaptation to nicotine and its metabolites, which lead to an initial lowering of BP, has already been studied by some authors; however, the mechanism remains unclear.^10-15^ Therefore, we hypothesized that the protective effect of light smoking is a consequence of nicotine exposure. As previously mentioned, and in contrast to the prohypertensive effects of carbon monoxide, nicotine promotes hypotension, an effect which is only detectable after years of intense exposure.^15^

It should be noted that previous studies have demonstrated a proportional increase in hypertension risk according to the magnitude of tobacco exposure, whether measured in time or amount of cigarettes.^17,18^ This data contrasts the nonlinear influence of the number of cigarettes on the BP of active smokers, as described in this paper. Therefore, we emphasized the variable impact of tobacco exposure on BP and its dependence on the form of exposure (active or passive).

Another interesting result was the hypertension effect measure of former smoker groups in comparison to that of current heavy smokers (≥ 1 pack/day). These data corroborate the findings of previous research, which, in addition to showing a link between higher tobacco loads and lower BP levels, demonstrated that regular smoking was significantly associated with lower SBP and DBP. In contrast, smoking cessation was associated with higher DBP levels in both groups compared to never-smokers. Among the possible explanations for the higher hypertension risk in former smokers, one of the hypotheses attributes the BP increase to the weight gain that usually follows smoking cessation.^19-21^ Such a theory is probably the most widely accepted, especially since weight gain itself is among the main risk factors for hypertension.^22^ Furthermore, there are two other possible reasons for this phenomenon, albeit not so robustly backed by literature: the hypothesis of masked hypertension in active smokers and reverse causality.^16,23^

Lastly, it is worth noting that the physiopathological mechanisms of nicotine and its metabolites, as well as the influence of carbon monoxide on the nitric oxide cycle and the overall cardiovascular damage caused by tobacco smoke, which are crucial to sustaining our findings, have already been extensively explained in the scientific literature.^3,6^

The main limitation of our study is its cross-sectional design, which prevented the establishment of a causal relationship between the never-smoker and passive-smoker group and hypertension. Thus, despite finding significant associations, we could not confirm a causal relationship between these variables, and it is therefore essential to conduct new longitudinal studies to explore this association in the Brazilian population.

Additionally, this study’s reliance on the VIGITEL survey is also a limitation due to the self-reported nature of the information, which may lead to underestimations compared with methods such as serum cotinine analysis and blood pressure measurements. Additionally, there are other limitations to this study, such as not questioning the duration of the participants’ exposure to tobacco, years since smoking cessation, hypertension severity, years since hypertension diagnosis, and prescription of antihypertensives. These data could enhance our understanding of how passive smoking influences the development and progression of hypertension.^24^ Moreover, as already mentioned in the methodology section, the COVID-19 pandemic during 2020 and 2021 restricted the survey’s timeframe, thereby reducing the sample size.

Among the strengths of the study is the fact that this study used a solid national database focused on investigating NCD, including all states’ capitals and Federal District data, with a sample of over 600 thousand people. To our knowledge, this is the largest study in Brazil on this subject and the first to use a national population-based sample. We believe that our findings can be extrapolated to other countries because of the biological plausibility of the associations analyzed. Furthermore, our data can be used to implement policies on indoor smoking behavior.

## Conclusion

Overall, this study found a statistically significant association between passive smoking and hypertension. Additionally, a comparison was made between the prevalence of hypertension in passive and active smokers. Finally, we highlight the importance of developing more longitudinal studies on this topic, aiming for prolonged and targeted follow-ups to comprehend better the BP changes caused by passive smoking. This knowledge is necessary to promote effective prevention and damage reduction in the context of smoking and passive smoking.
